# Supplementary material for: On the generalization limits of quantum generative adversarial networks with pure state generators
Source: Sci Rep. 2026 Jun 9;16:17845. doi: 10.1038/s41598-026-54900-7 (PMC13250101; doi:10.1038/s41598-026-54900-7)
Supplement: Supplementary file 1 — Supplementary Information. [file 41598_2026_54900_MOESM1_ESM.pdf]

# Supplementary Material for “On the Generalization Limits of Quantum Generative Adversarial Networks with Pure State Generators”

**Jasmin Frkatovic[1], Akash Malemath[1,4], Ivan Kankeu[1], Yannick Werner[1,2], Matthias Tschöpe[1], Vitor Fortes Rey[1,2], Sungho Suh[3], Paul Lukowicz[1,2], Nikolaos Palaiodimopoulos[1,2], and Maximilian Kiefer-Emmanouilidis[1,2,4,*]**

[1]Department of Computer Science and Research Initiative QC-AI, RPTU Kaiserslautern-Landau, Kaiserslautern, Germany
[2]Embedded Intelligence, German Research Center for Artificial Intelligence (DFKI), Kaiserslautern, Germany
[3]Department of Artificial Intelligence, Korea University, Seoul, Republic of Korea
[4]Department of Physics, RPTU Kaiserslautern-Landau, Kaiserslautern, Germany
[*]maximilian.kiefer@rptu.de

## ABSTRACT

We investigate the capabilities of Quantum Generative Adversarial Networks (QGANs) in image generations tasks. Our analysis centers on fully quantum implementations of both the generator and discriminator. Through extensive numerical testing of current main architectures, we find that QGANs struggle to generalize across datasets, converging on merely the average representation of the training data. When the output of the generator is a pure-state, we analytically derive a lower bound for the discriminator quality given by the fidelity between the pure-state output of the generator and the target data distribution, thereby providing a theoretical explanation for the limitations observed in current models. Our findings reveal fundamental challenges in the generalization capabilities of existing quantum generative models. While our analysis focuses on QGANs, the results carry broader implications for the performance of related quantum generative models.

## Supplementary Material

### Loss Plots

In Fig. 1(a) we plot the loss curve for the IQGAN model trained on class 3 of MNIST dataset. Figs. 1(b) and (c) display the corresponding loss curves for the QuGAN model trained on class 3 and on classes 3, 6, and 9, respectively. We observe that the pronounced oscillations in the QuGAN model hinder stable convergence, which has been reported prior by[1,2].

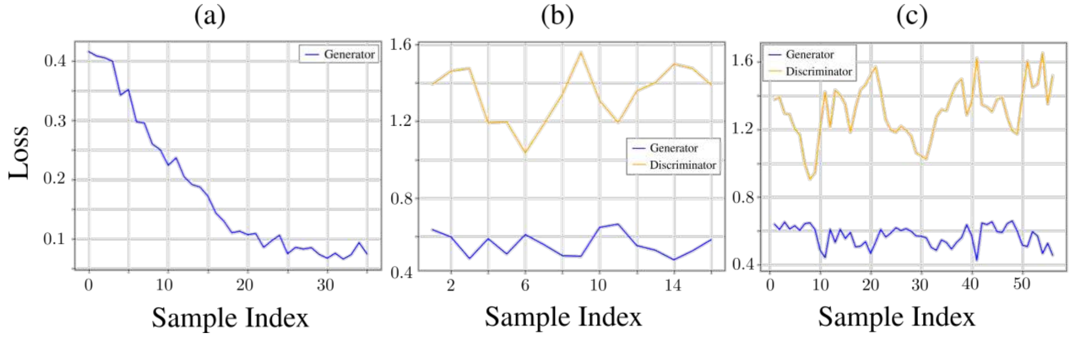


**Figure 1.** Loss curves for: (a) IQGAN and (b) QuGAN trained on class 3 of MNIST. (c) QuGAN trained on classes 3,6, and 9 of MNIST. Loss values were sampled every 40 batches throughout the training process.

### Limitations of a QGAN Generator

In classical GANs the generator receives noise vectors as input, enabling it to produce diverse samples from the target distribution. A key limitation that may hinder this process in variational quantum circuits (VQCs) is the absence of non-linear

activation functions, a consequence of the unitary nature of quantum operations. Non-linearities are essential for enhancing the expressivity of neural networks, and their absence in quantum models could restrict the generator's ability to model complex distributions.

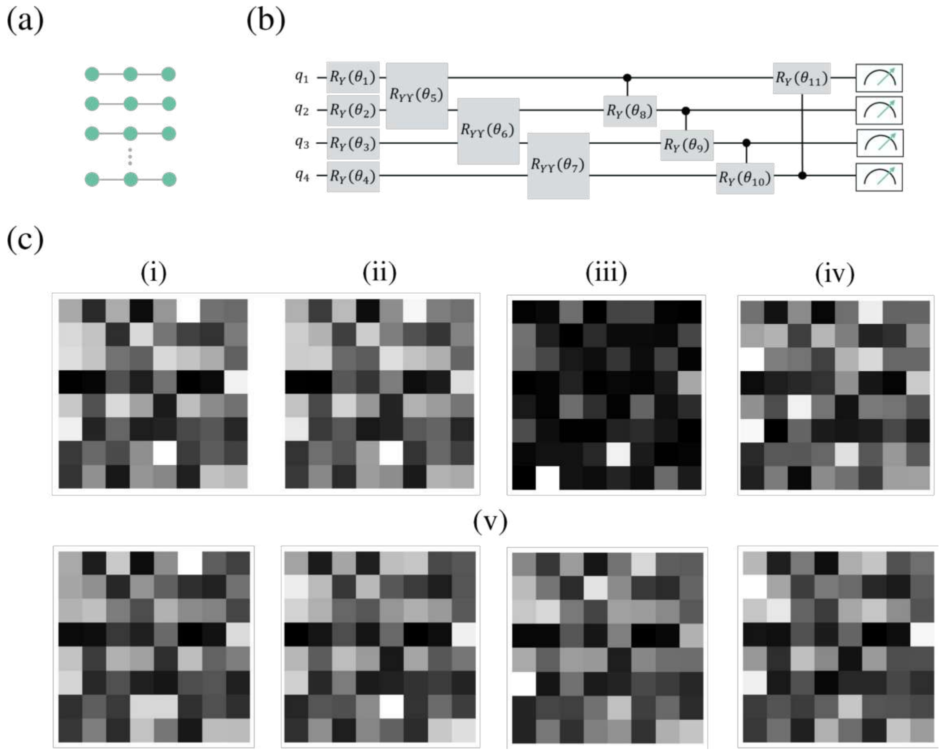


**Figure 2.** (a) Schematic of the classical neural network architecture. (b) Quantum generator circuit used in QuGAN. (c) Visualization of target and generated images: (i) Target image of size $8 \times 8$, with pixel values sampled from a uniform distribution over [0,1]; (ii) Output image generated by the trained classical neural network; (iii) Image generated by the QuGAN generator with 6 qubits and 4 layers; (iv) Image generated by the QuGAN generator with 12 qubits, including 6 ancillary qubits; (v) Sample outputs from the trained model in (c) for various random input vectors.

To highlight the importance of non-linearities, we designed a simple test comparing the effectiveness of a classical toy model of a neural network (NN) with the generator circuit used in QuGAN. In this setup, both models receive noise vectors as input at each epoch, with elements randomly sampled from a uniform distribution. The task is to reproduce a specific target image, where each pixel value is also randomly drawn from a uniform distribution.

As illustrated in the schematic of Fig. 2(a), the layers of the classical NN are not fully connected. On the contrary, each neuron is connected only to its corresponding neuron in the next layer. Additionally, the weights are fixed and set to 1, ensuring that the input values remain unchanged. The only trainable parameters are the biases, which are added to the inputs before being passed through a non-linear activation function applied to the hidden layer neurons. This deliberately constrained architecture is intended to isolate and examine the effect of non-linearity. As for the QuGAN generator—one layer of which is illustrated in Fig. 2(a)—we set the number of layers such that the total number of trainable parameters is approximately equal to that of the classical neural network (i.e. the number of nodes). However, we acknowledge that this does not constitute a strictly fair comparison, as the representational capacities and training dynamics of quantum and classical models differ significantly. In Fig. 2(c)(i) we present the target image. Adjacent to it, Fig. 2(c)(ii) shows of the classical neural network, and Fig. 2(c)(iii) displays the output of the QVC. We observe that even this highly constrained version of a classical neural network is able to closely approximate the target image, whereas the QVC clearly struggles to do so. A common approach to inducing non-linear transformations in quantum circuits involves introducing ancillary qubits, performing partial measurements, and tracing out the ancillary subsystem[3,4]. The non-linear effect can be amplified by increasing the number of ancillary qubits. As shown in Fig. 2(c)(iv), the generated output begins to approximate the target image more closely as the number of ancillary qubits approaches that of the generator's qubits (i.e., 12 qubits in total, with 6 ancillary). We also have to note that in this case the resulting generated state is not pure, therefore the limitations presented in Section "Fidelity Bounds in QGANs" do not apply. In this case, once the model is trained, providing noise vectors as input leads to the generation of output states that exhibit a

degree of variability (see Fig. 2(c)(iv)). However, our attempts to train generative variational circuits with the aforementioned characteristics on multiple classes of the MNIST dataset were unsuccessful.

## Training details

All models are trained using the Adam optimizer with a learning rate of 0.001 and a batch size of 32. Training is performed for 10 epochs, which we keep fixed since the generator loss and fidelity empirically converge within this range, with no noticeable improvement beyond that. For the QGAN setup, both the generator and discriminator are parametrized quantum circuits operating on $k$ qubits, where $k$ corresponds to the number of retained PCA components. Per iteration, the discriminator is trained using a real-data minibatch and 16 fake samples generated by the generator, while the generator is subsequently updated using 8 discriminator evaluations. After each epoch, a sample is generated by measuring the generator qubits, forming a $k$-dimensional vector that is mapped back to an image via inverse normalization and inverse PCA.

## Effect of Noise

In this section we discuss how noise on Noisy Intermediate-Scale Quantum (NISQ) devices impacts the performance of QGANs. Our numerical simulations in the main text focus on an idealized, noise-free setting in order to isolate fundamental limitations of the model class. If we consider noise, the model would never be able to reach the optimum unless the hardware noise $\rho_{\text{inf}}$ coincides with $\rho_{\text{data}}$. To see this we can account for noise in a general and analytically tractable manner. Here, we model the action of hardware noise on the generator output by an effective completely positive trace-preserving (CPTP) map with stationary state $\rho_\infty$. The noisy generator output can then be written as

$$\rho_G^{(\varepsilon)} = (1-\varepsilon)\,\rho_G + \varepsilon\,\rho_\infty,$$

where $\varepsilon \in [0,1]$ quantifies the noise strength. Writing $\rho_{\text{data}} - \rho_G^{(\varepsilon)} = (1-\varepsilon)(\rho_{\text{data}} - \rho_G) + \varepsilon(\rho_{\text{data}} - \rho_\infty)$, the reverse triangle inequality for the trace norm yields

$$\left\|\rho_{\text{data}} - \rho_G^{(\varepsilon)}\right\|_1 \geq \left|\varepsilon \left\|\rho_{\text{data}} - \rho_\infty\right\|_1 - (1-\varepsilon)\left\|\rho_{\text{data}} - \rho_G\right\|_1\right|.$$

Crucially, even in the ideal training limit $\rho_G \to \rho_{\text{data}}$, the inequality above remains constrained by an irreducible noise-induced contribution,

$$\left\|\rho_{\text{data}} - \rho_G^{(\varepsilon)}\right\|_1 \;\to\; \varepsilon \left\|\rho_{\text{data}} - \rho_\infty\right\|_1,$$

which vanishes only when the stationary noise state $\rho_\infty$ coincides with the data state $\rho_{\text{data}}$. Consequently, this limitation cannot be eliminated through further optimization of the generator parameters.

We therefore emphasize that our idealized simulations should be interpreted as providing upper bounds on achievable performance. The inclusion of realistic NISQ noise can only further restrict the attainable fidelity and, in extreme cases, render QGAN training ineffective altogether.

To complement the above analytical argument, we consider a representative example where we examine both QuGAN and IQGAN architectures on MNIST class 3 with $k = 4$ PCA components. Both models are trained in the presence of local depolarizing noise, where a depolarizing channel of probability $p$ is applied independently to each qubit.

After training at a fixed depolarizing probability, we repeatedly sample bitstrings from the noisy generator and compute the FID between the reconstructed noisy samples and the real MNIST class-3 images. The FID is evaluated over 100 independent realizations. In Fig. 3, we plot the mean FID as a function of the depolarizing probability, with error bars indicating the standard deviation at each point.

We observe that for QuGAN, the mean FID fluctuates as the depolarizing probability increases. Nevertheless, all values remain within a similarly poor range around the noiseless baseline. For IQGAN, the FID decreases more steadily as the depolarizing probability increases and eventually falls below the noise-free baseline. However, this should not be interpreted as the model learning the data distribution more accurately. Instead, stronger depolarizing noise drives the generated outputs toward increasingly average-like images that lie closer to the dominant modes of the dataset, thereby yielding slightly lower FID values. This behavior is consistent with the numerical findings presented in the main text when evaluating the performance of each model.

This numerical example is not intended as a realistic hardware benchmark, but rather as an illustration of the general point made above: namely, that model performance generally does not improve relative to the noise free case.

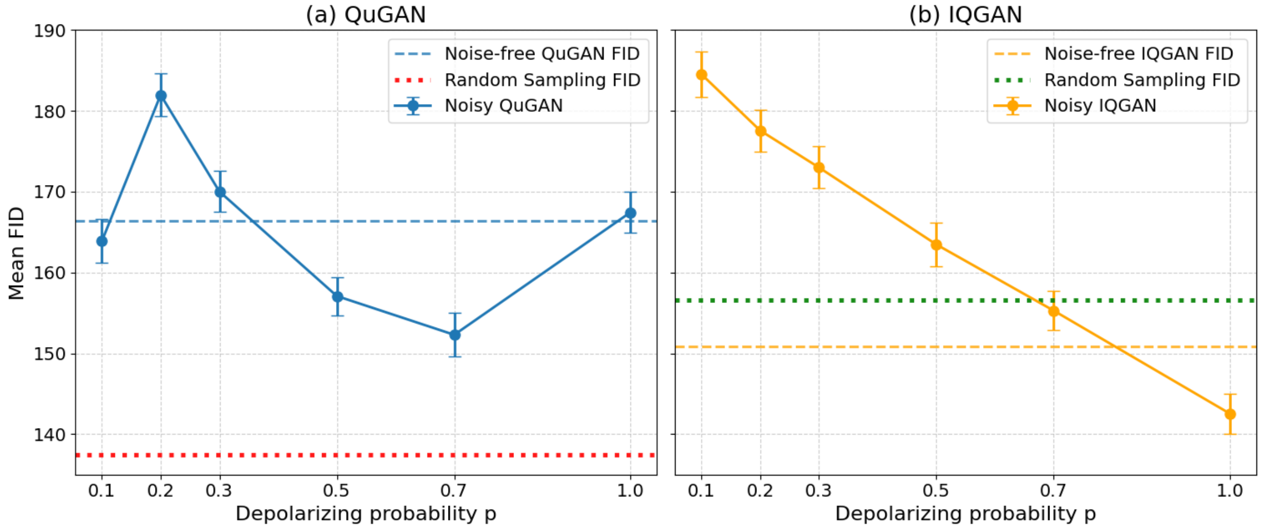


**Figure 3.** Mean FID scores as a function of depolarizing noise probability $p$ for (a) QuGAN and (b) IQGAN. Each point is computed by averaging over 100 realizations and the error bars correspond to the standard deviation. The dashed horizontal lines represent the FID values of the corresponding noise-free models and the dotted lines the FID values obtained by uniformly random sampling of latent bitstrings.

## References


1. C. Chu, G. Skipper, M. Swany, and F. Chen, *"IQGAN: Robust quantum generative adversarial network for image synthesis on NISQ devices"*, IEEE International Conference on Acoustics, Speech and Signal Processing (ICASSP) 1–5 (2023).
2. M. Y. Niu, A. Zlokapa, M. Broughton, S. Boixo, M. Mohseni, V. Smelyanskyi, and H. Neven, *"Entangling quantum generative adversarial networks"*, Phys. Rev. Lett. **128**, 220505 (2022).
3. H.-L. Huang, Y. Du, M. Gong, Y. Zhao, Y. Wu, C. Wang, S. Li, F. Liang, J. Lin, Y. Xu, and others, *"Experimental quantum generative adversarial networks for image generation"*, Phys. Rev. Appl. **16**, 024051 (2021).
4. I. Cong, S. Choi, and M. D. Lukin, *"Quantum convolutional neural networks"*, Nat. Phys. **15**, 1273–1278 (2019).
